# MACC1 as a Prognostic Biomarker for Early-Stage and AFP-Normal Hepatocellular Carcinoma

**DOI:** 10.1371/journal.pone.0064235

**Published:** 2013-05-23

**Authors:** Chan Xie, Jueheng Wu, Jingping Yun, Jiaming Lai, Yunfei Yuan, Zhiliang Gao, Mengfeng Li, Jun Li, Libing Song

**Affiliations:** 1 Department of Infectious Diseases, the Third Affiliated Hospital of Sun Yat-sen University, Guangzhou, Guangdong Province, China; 2 State Key Laboratory of Oncology in Southern China, Department of Experimental Research, Cancer Center, Sun Yat-sen University, Guangzhou, Guangdong Province, China; 3 Department of Microbiology, Zhongshan School of Medicine, Sun Yat-sen University, Guangzhou, Guangdong Province, China; 4 Department of Pathology, Cancer Center, Sun Yat-sen University, Guangzhou, Guangdong Province, China; 5 Department of Hepatobiliary Surgery, the First Affiliated Hospital of Sun Yat-sen University, Guangzhou, Guangdong Province, China; 6 Department of Hepatobiliary Surgery, Cancer Center, Sun Yat-sen University, Guangzhou, Guangdong Province, China; 7 Key Laboratory of Tropical Disease Control, Ministry of Education, Sun Yat-sen University, Guangzhou, Guangdong Province, China; 8 Department of Biochemistry, Zhongshan School of Medicine, Sun Yat-sen University, Guangzhou, Guangdong Province, China; National Cancer Institute, United States of America

## Abstract

**Background:**

The metastasis-associated in colon cancer 1 gene (MACC1) has been found to be associated with cancer development and progression. The aim of this study was to investigate the prognostic value of MACC1 in early-stage and AFP-normal hepatocellular carcinoma (HCC).

**Methods:**

mRNA and protein levels of MACC1 expression in one normal liver epithelial cells THLE3 and 15 HCC cell lines were examined using reverse transcription-PCR and Western blot. MACC1 expression was also comparatively studied in 6 paired HCC lesions and the adjacent non-cancerous tissue samples. Immunohistochemistry was employed to analyze MACC1 expression in 308 clinicopathologically characterized HCC cases. Statistical analyses were applied to derive association between MACC1 expression scores and clinical staging as well as patient survival.

**Results:**

Levels of MACC1 mRNA and protein were higher in HCC cell lines and HCC lesions than in normal liver epithelial cells and the paired adjacent noncancerous tissues. Significant difference in MACC1 expression was found in patients of different TNM stages (*P*<0.001). Overall survival analysis showed that high MACC1 expression level correlated with lower survival rate (*P* = 0.001). Importantly, an inverse correlation between MACC1 level and patient survival remained significant in subjects with early-stage HCC or with normal serum AFP level.

**Conclusions:**

MACC1 protein may represent a promising biomarker for predicting the prognosis of HCC, including in early-stage and AFP-normal patients.

## Introduction

Primary hepatocellular carcinoma (HCC) is the fifth most commonly diagnosed cancer and the third most common cause of cancer mortality worldwide [1]. HCC carcinogenesis involves aberrant changes in multiple molecular pathways and genetic as well as epigenetic factors, which consequently results in malignant transformation and progression of HCC [2], [3]. Unfortunately, the long-term prognosis of patients with HCC remains unsatisfactory in spite of recent advances in surgical techniques and medical management. No specific signature of liver cancer gene expression has been reported to allow for patient-tailored therapy strategies. Hence, it is of great clinical value to further identify effective early markers for the diagnosis and prognosis of the disease as well as novel therapeutic targets.

Clinically, prediction of prognosis plays an essential role in the assessment of HCC patients and optimal therapeutic options. Currently, both tumor- (e.g., alpha fetal protein (AFP), tumor size and portal vein thrombosis) and cirrhosis-based (mainly, the Child–Pugh class) parameters are commonly employed to predict patient survival [4]. While tremendous effort has been made in identifying effective indicators for the prognostic prediction of HCC, the availability of clinically applicable biomarkers remains limited. Prediction of clinical prognosis of HCC is still reliant on conventional pathologic variables such as tumor size, tumor grade, lymph node, and distal metastasis status [5]. Furthermore, as the number of HCC patients who are in early stages of the disease, bear small tumor nodules (<3 cm), or display normal serum level of AFP is increasing, finding new prognosis prediction biomarker for these patients represents a major challenge in the clinic.

MACC1, a novel regulator of tumor growth and metastasis, has recently been indicated as a potential prognostic factor for metastatic disease in HCC [6], [7], lung adenocarcinoma [8], gastric carcinoma [9] and colorectal cancer [10], [11]. Expression of MACC1 was found to be significantly upregulated in malignant tissues compared to normal tissues or adenomas, and induction of MACC1 might occur at the crucial step of transition from the benign to the malignant phenotype and can be allocated to the adenoma–carcinoma sequence for colon cancer [10]. It is of note that hepatocyte growth factor (HGF) receptor, MET, is a transcriptional target of MACC1. MACC1 enhances proliferation and invasion as well as HGF-induced scattering of colon cancer cells in vitro and promotes tumor growth and metastasis of xenografted tumors in mouse [12]. Transduction of MACC1 mutants with the SH3 domain or the proline-rich motif deleted in colon cancer cells abrogated the above function of MACC1 [13].

It has been reported that MACC1 mRNA expression in HCC tissue was significantly higher than that in nonmalignant tissues, and high MACC1 mRNA expression correlated with more aggressive behavior in terms of shorter overall survival (OS) and disease-free survival [7]. However, the protein expression level of MACC1 in HCC was not investigated in these studies. In our current report, immunohistochemical analysis was performed to investigate the potential prognostic utility of MACC1 in both a test and a validation cohort of HCC in comparison with non-neoplastic liver tissues, including HCC patients with normal serum AFP level of early stage. Our data suggest that MACC1 might represent a valuable prognostic biomarker for HCC.

## Materials and Methods

### Normal Liver Samples

Three normal liver samples were collected from patients undergoing resection of hepatic hemangiomas at the Department of Hepatobiliary Surgery, the First Affiliated Hospital of Sun Yat-sen University with prior written informed consents from the patients and approval from the Institutional Research Ethics Committees of Sun Yat-sen University and its First Affiliated Hospital.

### Liver Cancer Tissue Specimen

Formalin-fixed, paraffin-embedded primary HCC specimens obtained from 308 patients, who underwent initial surgical resection between March 1995 and August 2008, were randomly selected from the archives of the Sun Yat-sen University Cancer Center (Guangzhou, China). Fresh HCC tissue samples, together with their paired adjacent non-cancerous tissues from each patient, were collected from HCC curative resection surgery, snap frozen and stored at −80°C until use for experimental purposes. For the use of these clinical materials for research purposes, prior patients’ written informed consents and approval from the Institutional Research Ethics Committee of Sun Yat-sen University Cancer Center were obtained. This cohort of patients with HCC included 249 (80.8%) men and 59 (19.2%) women, with a median age of 48.6 years, and their clinico-pathological characteristics are summarized in Table 1. Tumor stages were defined according to the 2002 American Joint Committee on Cancer/International Union against Cancer tumor/lymph node metastasis/distal metastasis (TNM) classification system. Hepatitis B virus (HBV) infection was diagnosed when HBV surface antigen (HBsAg) was detected by ELISA in the serum.

**Table 1:** Clinicopathological characteristics of clinical samples and expression of MACC1 in liver cancer.

Characteristics	No. patients	(%)
**Age** (**years**)		
≤50	182	(59.1)
>50	126	(40.9)
**Gender**		
male	249	(80.8)
female	59	(19.2)
**TNM classification**		
I	26	(8.4)
II	195	(63.3)
III	61	(19.8)
IV	26	(8.4)
**HBsAg**		
positive	258	(88.1)
negative	35	(11.9)
**AFP**		
>400 **ng/ml**	107	(35.8)
≤400** ng/ml**	192	(64.2)
**Tumor size**		
>3 cm	260	(85.0)
≤3 cm	46	(15.0)
**Tumor number**		
>1	102	(33.3)
= 1	204	(66.7)
**Vital status** (**at follow-up**)		
Alive	161	(52.3)
Death due to liver cancer cause	147	(47.7)
**Expression of MACC1**		
Low expression	182	(59.1)
High expression	126	(40.9)

### Cell Lines

HCC cell lines, including QGY-7703, QGY-7701, SMMC-7721, HepG2, Hep3B, PLC/PRF5, HuH7, HepG-2215, HCCC-9810, Bel-7402, Bel-7404, HCCLM3, HCCLM6, MHCC97L and MHCC97H, were grown in Dulbecco's modified Eagle's medium (DMEM) (Invitrogen, Carlsbad, CA) supplemented with 10% fetal bovine serum (FBS) (Invitrogen). Immortalized normal liver epithelial cells, THLE3, were maintained in bronchial epithelial growth medium (Lonza Cologne GmbH, Walkersville, MD), with 5 ng/ml epidermal growth factor, 70 ng/ml phosphoethanolamine and 10% FBS, at 37°C in a humidified atmosphere containing 5% CO2. Primary human hepatocytes (PHH) were separated as previously reported [14].

### Immunohistochemistry (IHC)

The IHC procedure for MACC1 and scoring of MACC1 expression were performed as previously reported [15]. IHC staining was quantitatively analyzed with the AxioVision Rel.4.6 computerized image analysis system assisted with the automatic measurement program (Carl Zeiss, Oberkochen, Germany). Briefly, the stained sections were evaluated at 200× magnification, and ten representative staining fields of each section were analyzed to verify the Mean Optical Density (MOD), which represented the strengths of staining signals as measured per positive pixels. The MOD data were statistically analyzed using *t*-test to compare the average MOD difference between different groups of tissues, and *P*<0.05 was considered significant.

### RNA Extraction, Reverse Transcription (RT) and Real-time PCR

Total RNA from cultured cells was extracted using the Trizol reagent (Invitrogen, Carlsbad, CA) as the manufacturer instructed. cDNAs were amplified and quantified in ABI Prism 7500 Sequence Detection System (Applied Biosystems, Foster City, CA) using dye SYBR Green I (Invitrogen, Carlsbad, CA). The MACC1 primers designed using the Primer Express v 2.0 software (Applied Biosystems) are provided as following: forward: 5′- TTCTTTTGATTCCTCCGGTGA -3′ and reverse: 5′-TTCTTTTGATTCCTCCGGTGA -3′. Expression data were normalized to the geometric mean of housekeeping gene GAPDH (forward: 5′-ACCACAGTCCATGCCATCAC-3′ and reverse: 5′-TCCACCACCCTG TTGCTGTA -3′) to control the variability in expression levels and calculated as 2^−[(*C*^ *t* ^of MACC1) – (*C*^ *t* ^of *GAPDH*)]^, where C_t_ represents the threshold cycle for each transcript.

### Western Blot Analysis

Total protein was prepared using the cell total protein extraction kits according to the manufacturer’s instruction (Millipore, Billerica, MA). Western blot was performed according to standard methods, using an anti-MACC1 antibody (Prosci, Poway, CA). Blotted membranes were stripped and re-probed with an anti-GAPDH antibody (Sigma, Saint Louis, MI) as a loading control.

### Statistical Analysis

All statistical analyses were carried out using the SPSS v. 13.0 statistical software packages (SPSS, Chicago, IL). Comparisons between groups for statistical significance were performed with a two-tailed paired Student’s *t* test. The relationship between MACC1 expression and clinic pathological characteristics was analyzed by the chi-square test. Survival curves were plotted by the Kaplan-Meier method and compared using the log-rank test. Independent prognostic factors were estimated by the Cox proportional hazards stepwise regression model. Spearman’s correlation test was applied to analyze the correlation. All *P* values were two-sided. A *P* value of less than 0.05 was considered statistically significant in all cases.

## Results

### Upregulation of MACC1 in HCC Cell Lines and Liver Cancer Lesions

**Figure 1:**
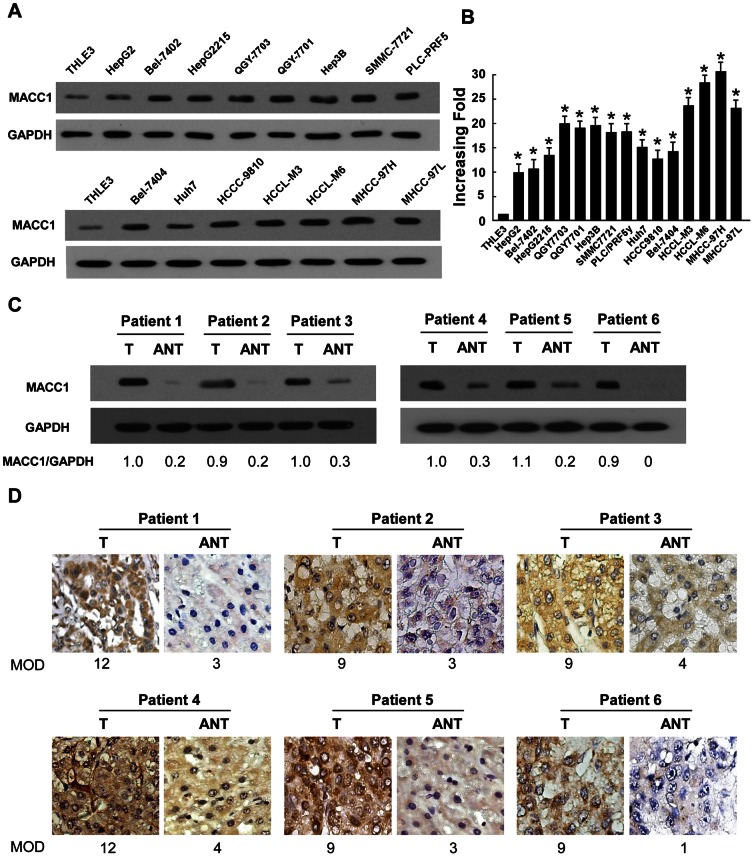
Expression of MACC1 is elevated in HCC. (**A-B**) protein (A) and mRNA (B) expression of MACC1 in THLE3 and 15 cultured liver cancer cell lines. GAPDH was used as a loading control. (**C-D**) Western blot (C) and IHC (D) analysis of MACC1 protein in each of the primary liver cancer tissue (T) and adjacent non-cancerous tissues (ANT) samples taken from the same patient. Error bars represent mean ± SD from three independent experiments. * *P*<0.05.

Western blot and real-time PCR analyses revealed higher levels of MACC1 expression in all fifteen HCC cell lines than that in THLE3 (Figure 1A and B). The MACC1 expression level of PHH and two normal liver tissues was as low as in THLE3 (Figure S1). To determine whether MACC1 upregulation found in liver cancer cell lines was clinically relevant, Western blot analysis was performed with 6 paired HCC tissues and non-cancerous tissues adjacent to HCC tumors, with each pair taken from the same patient. As shown in Figure 1C, MACC1 protein was overexpressed in all six examined primary HCC samples, displaying more than 2-fold increase of MACC1 expression as compared that in the adjacent non-cancer tissue samples. In agreement with the result of Western blot assay, immunohistochemical analysis also showed MACC1 upregulation in HCC lesions (Figure 1D). These results indicate that MACC1 might represent a biological marker clinically suggestive of a presence of HCC in human.

### Association between MACC1 Expression and Clinical Features of Liver Cancer

**Figure 2:**
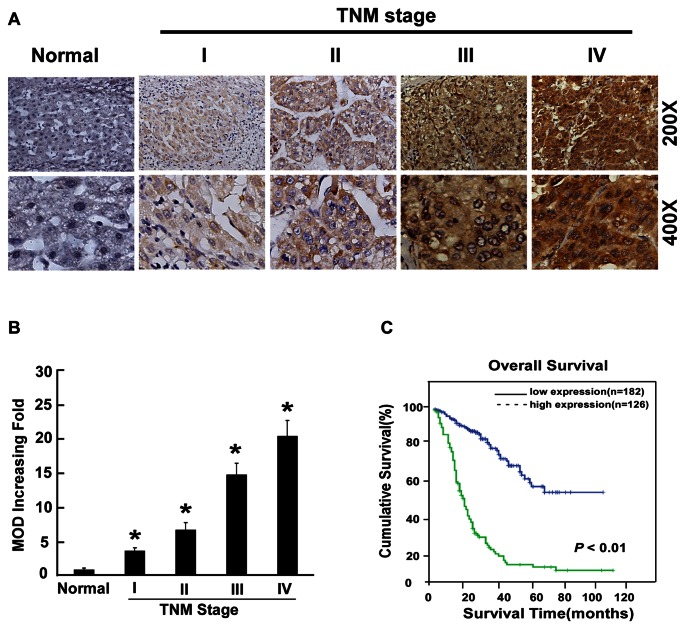
Overexpression of MACC1 in archived HCC. (**A**) Representative IHC analyses of MACC1 expression in normal liver tissue (Normal) and HCC specimens of different TNM stages. (**B**) Statistical quantification of the average MOD values of MACC1 staining between normal liver tissue (Normal) and HCC specimens of different TNM stages. The mean absorbance (MOD) of MACC1 staining increases as HCC progresses to a higher clinical stage. Error bars represent mean ± SD from three independent experiments. * *P*<0.05. (**C**) Kaplan–Meier curves with univariate analyses (log-rank) for patients with low- versus high-MACC1 expression.

To determine the clinical significance of MACC1, the correlation between MACC1 overexpression and the clinicopathological features of HCC was investigated in a retrospective cohort of 308 HCC cases by IHC, including 26 cases of stage I (8.4%), 195 cases of stage II (63.3%), 61 cases of stage III (19.8%) and 26 cases of stage IV liver cancers (8.4%), based on the TNM staging. In the cohort, 88.1% patients had HBV infection. MACC1 expression in 308 enrolled patient samples was determined as strong (score >6) in 126 cases (40.9%) and weakly positive or negative (scored 0–6) in 182 cases (59.1%) (Table 1). As shown in Figure 2A, the immunoreactivity of MACC1 was detected at variable levels and localized in the cellular cytoplasm. The MACC1 protein expression was generally negative in normal liver tissues, weak in early stage HCC (TNM stages I and II) and strong in later stage HCC (TNM stages III and IV) tissues. Quantitative analysis of the IHC staining indicated that MACC1 expression in clinical stage I–IV primary tumors was statistically higher than that in normal liver tissues (*P*<0.05, Figure 2B). The IHC analysis summarized in Table 2 showed that MACC1 was drastically upregulated in HCC lesions of patients of later stages of HCC (TNM stages III-IV) as compared with that in the early-stage HCC. Moreover, Spearman analysis revealed correlations between MACC1 and TNM classification (*P*<0.001), survival time (*P* = 0.001), vital status (*P*<0.001) and gender (*P* = 0.042) (Table 3). No significant associations between expression of MACC1 and other clinicopathological parameters such as age, hepatitis B surface antigen (HBsAg) status, tumor size and number of tumor nodules (Table 3), further suggesting a strong correlation of MACC1 expression with HCC clinical staging and patient survival.

**Table 2:** Correlation between MACC1 expression and clinicopathologic characteristics of liver cancer patient.

Characteristic	MACC1Protein level		Chi-squaretest
	Low	High	*P* value
**Age** (**>50 versus** **≤50 years**)	79/103	47/79	*0.292*
**Sex** (**M versus F**)	149/33	100/26	*0.659*
**TNM stage** (**I/II** **versus III/IV**)	151/31	70/56	*0.000*
**Tumor size** (**>3 cm** **versus ≤3 cm**)	148/33	112/13	*0.073*
**AFP** (**≥400 ng/ml** **versus <400 ng/ml**)	64/111	42/81	*0.713*
**Tumor number**(**>1 versus 1**)	57/124	45/80	*0.460*
**Vital status** (**Alive/Death**)	136/46	25/101	*0.000*
**HBsAg positive/negative**	151/19	107/16	*0.716*

**Table 3:** Spearman analysis of correlation between MACC1 and clinicopathological characteristics.

Variables	MACC1 expression level	
	Spearman Correlation	*P* Value
**Survival time**	**−0.182**	***0.001***
**Vital status**	**0.487**	***0.000***
**HBsAg**	**0.032**	***0.582***
**Age**	**−0.026**	***0.643***
**TNM**	**0.469**	***0.000***
**AFP**	**0.042**	***0.473***
**gender**	**0.116**	***0.042***
**Tumor number**	**0.042**	***0.466***
**Tumor size**	**0.083**	***0.146***

### Univariate and Multivariate Analyses of the Prognostic Power of MACC1

To identify variables with potential prognostic significance in HCC patients, univariate analysis for each variable was performed in relation to the survival time. In our univariate analysis, stepwise inclusion of variables in the model showed that significant prognostic factors included MACC1 level, tumor size and TNM classification. Moreover, multivariate analysis also demonstrated that TNM staging, tumor size and MACC1 expression level are indeed predictive of the overall survival (OS) of HCC patients (Table 4).

**Table 4:** Univariate and multivariate analyses of various prognostic parameters in patients with liver cancer by Cox-regression analysis.

	Univariate analysis			Multivariate analysis		
	Relative risk	95% confidence interval	*P*	Relative risk	95% confidence interval	*P*
**TNM stage**	1.994	1.601–2.482	*0.000*	1.494	1.199–1.861	*0.000*
**Tumor size**	1.053	1.017–1.090	*0.004*	1.048	1.012–1.087	*0.009*
**MACC1**	5.132	3.923–7.978	*0.000*	4.823	3.257–6.893	*0.000*

### Prognostic Values of MACCs in Different HCC Subgroups

To further demonstrate the value of MACC1 expression in predicting survival of HCC patients, multiple analysis methods were performed in this study. Firstly, as shown in Figure 2C, Kaplan-Meier and log-rank survival tests suggested that low- and high-MACC1 expression in HCC patients were associated with different survival time, with the OS of patients expressing low MACC1 in their HCC lesions surviving much longer that those with high MACC1 expression (*P*<0.001). Interestingly, these patients with different OS could not be distinguished by conventional AFP test. While for the whole study cohort, the OS rates at 1,3, and 5 years, respectively, were 81%, 43% and 17%, patients with high MACC1 protein levels had a significantly lowered 1,3, and 5-year survival rate than those with low MACC1 protein levels (36%, 11%, 10% vs 87%, 69%, 53%, respectively, *P*<0.05).

**Figure 3:**
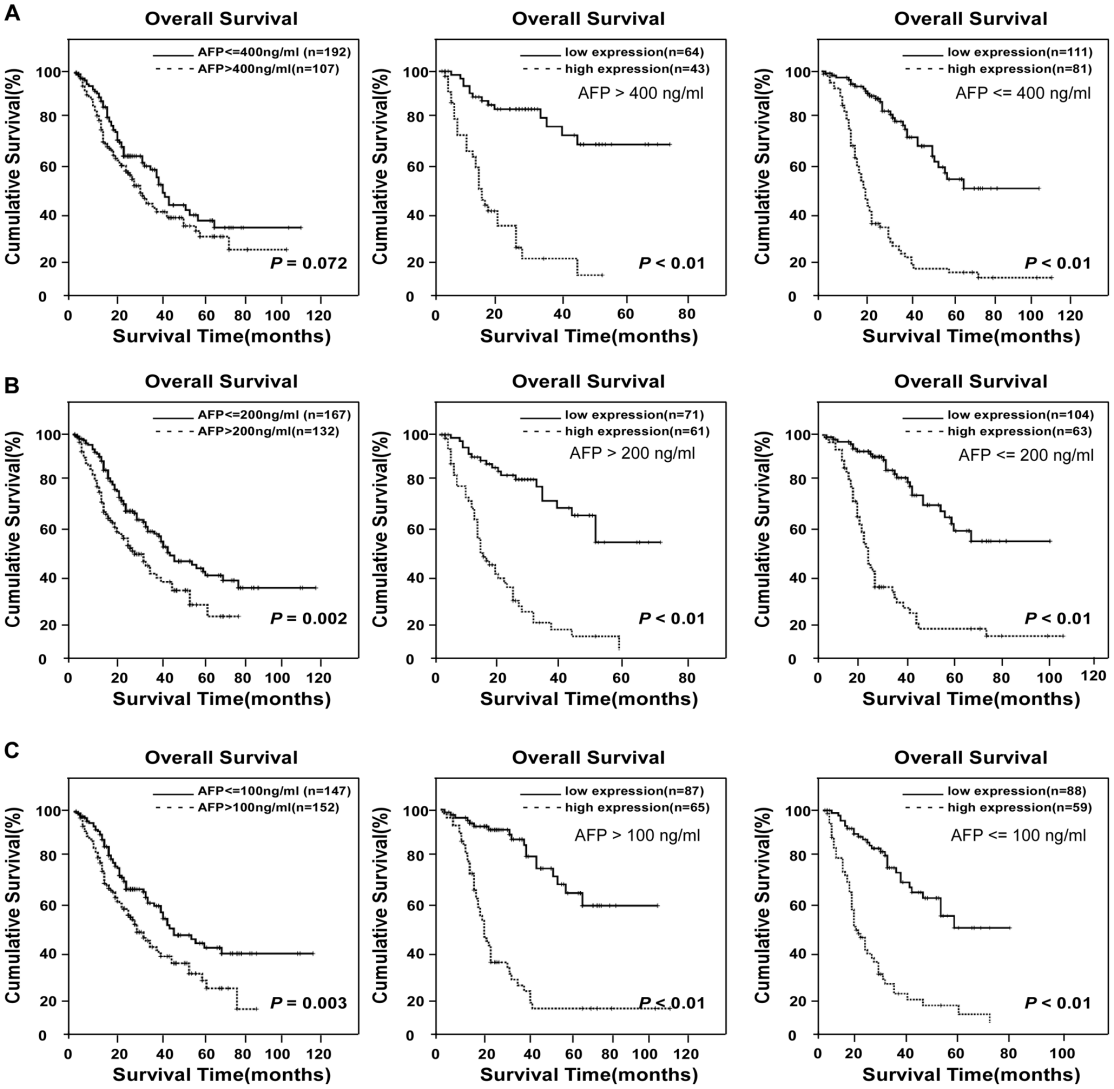
Kaplan-Meier analysis of OS in 308 patients based on MACC1 expressions in HCC subgroups of different AFP levels. (**A**) Using AFP level (400 ng/ml) as the cut-off could not separate patients with different OS rates in the study cohort (left). By contrast, MACC1 expression level predicted different OS rate in the subgroup of AFP≤400 ng/ml and AFP>400 ng/ml. (**B**) OS rate of patients with AFP≤200 ng/ml and AFP>200 ng/ml, respectively. (**C**) OS rates of patients with high- or low-MACC1 expression when further divided into AFP≤100 ng/ml and AFP>100 ng/ml subgroups.

**Figure 4:**
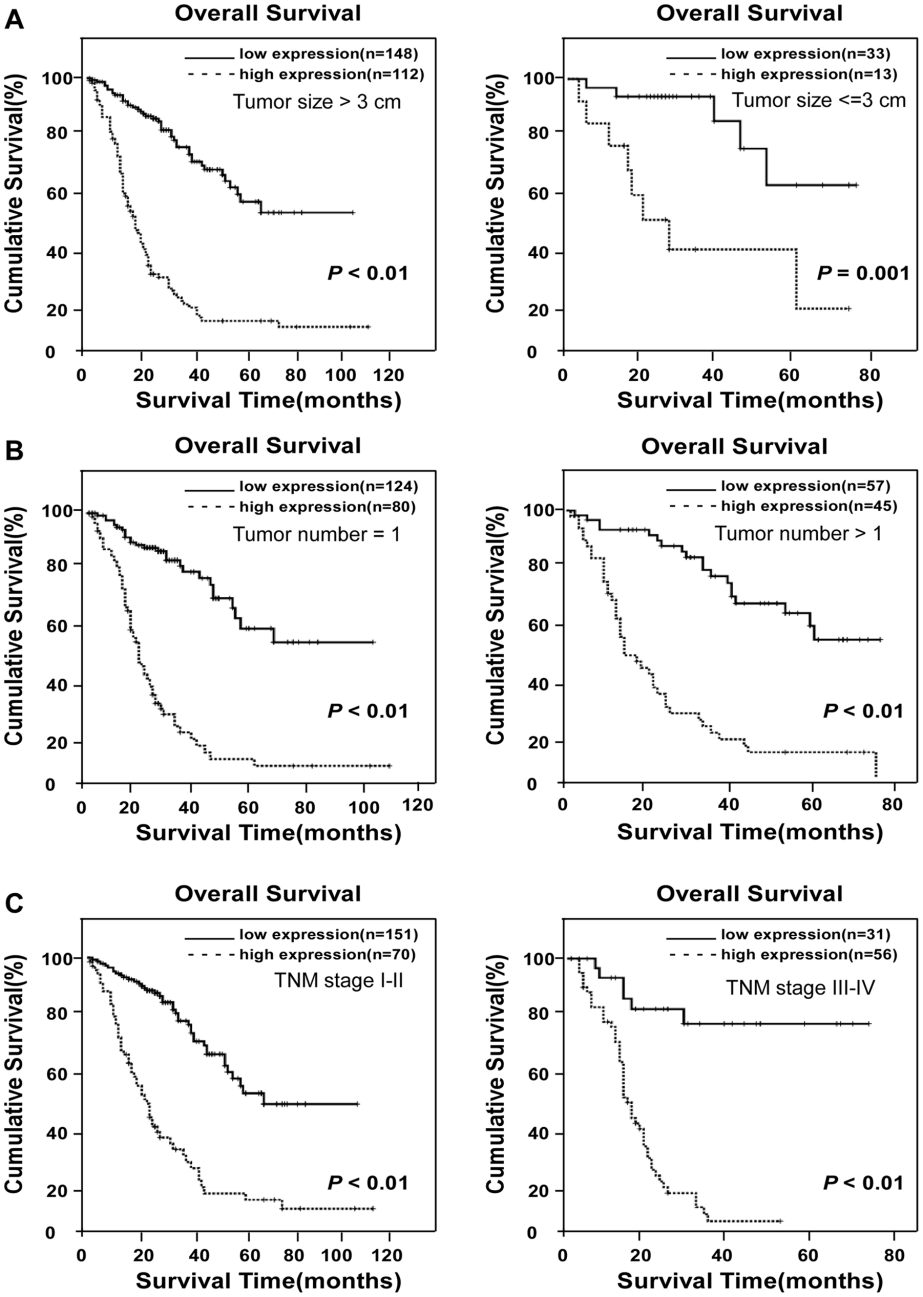
Kaplan-Meier analysis of OS in 308 patients based on MACC1 expressions in HCC clinical subgroups. (**A**) Statistical significance of the difference between curves of MACC1 high-expressing and low-expressing patients was compared in large HCC lesion (diameter >3 cm, left panel) and small HCC lesion (diameter ≤3 cm, right panel) patient subgroups. (**B**) OS in patients with single tumor lesion and multi-tumor lesion. (**C**) OS rates in patients subgrouped into TNM stages I-II (left) and TNM stages III-IV (right) as differentiated by high- or low-MACC1 expression.

A validation cohort was employed to further evaluate the prognostic value of MACC1 for specific subgroups of patients. As shown in Figure 3, the MACC1 levels were significantly associated with OS in patients with early-stage HCC, and such a predictive power was also observed in patients without AFP elevation. In the subgroup of patients with low AFP (≤400 ng/ml), MACC1-low expression was associated with a 5-year OS rate of 64%, in contrast to 19% for the high-MACC1 group (*P*<0.001, Figure 3A). Then we used different cutoff levels of AFP (400 ng/ml, 200 ng/ml and 100 ng/ml, respectively) to subgroup the 308 HCC patients and evaluated the prognostic significance of MACC1 in the patient subgroups. Our data suggested that AFP cut-off values of 200 ng/ml or 100 ng/ml) were significantly predictive of patient survival, whereas AFP = 400 ng/ml was not a prognostic cut-off. However, in all above HCC patient subgroups, the level of MACC1 was more sensitive to predict the prognosis of HCC patients than AFP. As shown in the subgroup of AFP level less than 200 or 100 ng/ml, MACC1 was able to separate patients with different OS rates as well (Figure 3B and 3C). In another group of HCC patients whose survival has been difficult to predict in the clinic, namely, those with tumor sizes smaller than 3 cm in diameter, the 5-year survival rate was 40% in the low MACC1 group, as opposed to 15% for patients exhibiting high MACC1 expression (*P = *0.002, Figure 4A). In the clinical subgroup with one single HCC tumor nodule, the 5-year survival rates were 54% and 13%, respectively, for low- or high-MACC1 expression patients (*P*<0.001, Figure 4B). In early-stage HCC patients (TNM stages I-II), low-MACC1 expression patients revealed a 5-year survival rate of 57%, whereas the survival rate decreased to 14% in the high MACC1 group (*P*<0.001, Figure 4C). Taken together, therefore, our data suggest a potentially promising prognostic value of MACC1 for HCC patients in various clinical subgroups that otherwise could have been difficult for survival prediction (Table 4).

## Discussion

In the present study, a cohort of patients (n = 308) were examined for MACC1 expression. Consistent results derived from three different assays, namely, Real-time PCR, blot analysis and IHC, strongly suggest a correlation between MACC1 level and the clinical outcome of HCC. Of note, such a correlation has been validated in an independent cohort. Identification of MACC1 as a prognostic biomarker for HCC in our present study provides new opportunities in the clinic for prediction of patient survival. The finding that high MACC1 expression level is present in HCC cell lines and clinical lesions, as well as its correlation with the clinical staging of HCC, laid a foundation for developing this immunologically and pathologically detectable molecule into a clinically applicable approach to improved patient management based on more accurate judgment on disease prognosis.

Previously, correlation of increased MACC1 expression with vascular invasion and serum AFP level was investigated in HCC [6]. The number of HCC patients included, however, was relatively small (n = 30) in the study, and the clinical predictive value for patients’ survival was not investigated. In another study, 128 HCC patients were analyzed for MACC1 mRNA levels, exhibiting significantly higher MACC1 mRNA in HCC lesions than that in normal liver tissue and a correlation between high MACC1 mRNA expression and reduced patient survival [7]. However, the study only detected MACC1 mRNA but left the protein level of MACC1 uninvestigated. To address these issues, our current study employed large numbers of patients in independent cohorts (n = 308). The consistency of the results of three assay methods (RT-PCR, immunoblotting and immunohistochemistry) and the repeat validations in the test as well as the independent validation cohort, provide with essential, reliable evidence for the clinical significance of MACC1 as a prognostic biomarker for HCC.

Serologic biomarkers, including AFP, are employed in the clinic for HCC screening and as an important predictor for patient survival after tumor resection [16]. The diagnostic sensitivity of AFP for early HCC, however, is only 39–64% when used alone, leading to the unsatisfying reality that a large number of HCC patients without AFP elevation are missed and subsequently progress to late stage-HCC before becoming clinically symptomatic and detectable [17]. Due to its low sensitivity in identifying new HCC cases that have not been detected by imaging technology previously, AFP has been shown to be only marginally effective in specific patient populations [17]. Indeed, only 62.1% of HCC patients in our study cohorts are AFP-positive. By contrast, in the patient group in which AFP level was not predictive for prognosis, MACC1 appeared to be indicative of survival time lengths that are differential among the patients. Thus, our study has exhibited the potential value of MACC1 in predicting patient survival in subgroups with normal AFP levels or in the early-stage HCC group, which would have been difficult using currently clinically available surrogate biomarkers.

It is widely recognized that early diagnosis and treatment are key to better clinical outcome of HCC patients. Using biomarkers to identify patients with a highest risk of developing worse prognosis may thus reduce mortality and medical costs. It is particularly noteworthy that in our test cohort, the patients with early HCC (TNM stages I-II) display a significantly higher levels (5–10 folds increase) of MACC1 in the HCC lesions than that in the normal liver tissue, and as the disease progresses to later stages, the MACC1 level increases further. In our validation cohort, early-stage (TNM stages I-II, tumor size less than 3 cm, single tumor nodule) HCC patients with high level of MACC1 protein immunostained also display a relative low OS than those diagnosed with late-stage HCC who carry high levels of MACC1 expression. In consistence with our observation, MACC1 has been mechanistically shown to induce cell proliferation, invasion and metastasis *in vivo* [12]. High potential of vascular invasion and metastasis is often the main biological basis for the poor prognosis of HCC [18], [19]. In this context, MET, as one of the key players in the processes of cell dissociation, angiogenesis and cell migration in HCC [20], has been shown to be a transcriptional target of MACC1 [11]. Thus, these characters of MACC1 thus warrant efforts to further explore the potential of MACC1 to become a promising biomarker for identifying patients with poor prognosis after surgical excision in early stages. Inclusion of metastatic cases in future studies will help address whether a high-level expression of MACC1 in early-stage HCC patients may have the potential to progress to poor survival.

The International Union against Cancer’s TNM staging is one of the most prevalent systems to classify HCC and used to predict patient prognosis. Although the TNM system has successfully graded patients in relevance to their prognosis according to the clinicopathological variables (e.g., tumor size, tumor number, lymph node metastasis and distant metastasis), it is still limited in providing predictive information key to determining therapeutic strategies in subgroups of HCC patients. For example, identification of patients with poor prognosis in early-stage HCC remains highly challenging. Thus, our current finding has provided evidence that the positive expression of MACC1 in HCC may be important for the detection of an aggressive phenotype or a phenotype predicting poor prognosis. We believe that the use of MACC1 protein, as examined by IHC, as a diagnostic biomarker of HCC could improve the prospects of the early detection of HCC, and that an improved rate of detection would have important prognostic implications for patients with HCC. It is necessary to point out that our current study is of the retrospective nature and the number of patients with HCC lesions smaller than 3 cm was small. Clearly, further prospective studies designed to include a larger number of HCC lesions smaller than 3 cm and patients with metastasis are needed to validate the conclusions of this study. Moreover, it would be of great clinical value to determine whether serologic MACC1 can be of diagnostic or prognostic significance.

## Supporting Information

Figure S1 **Expression of MACC1 is elevated in normal liver cell and tissues.** Western blot analysis of MACC1 expression in THLE3 cells, PHH cells and two normal liver tissues. GAPDH was used as a loading control.
(TIF)
