# Supplementary figures and images for: MACC1 as a Prognostic Biomarker for Early-Stage and AFP-Normal Hepatocellular Carcinoma
Source: PLoS One. 2013 May 23;8(5):e64235. doi: 10.1371/journal.pone.0064235 (PMC3662710; doi:10.1371/journal.pone.0064235)

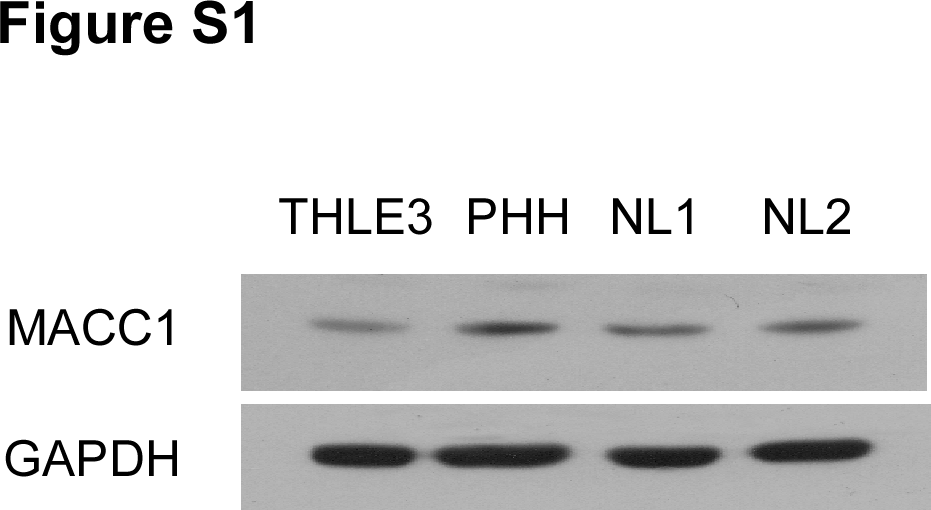

Supplement: Figure S1 — Expression of MACC1 is elevated in normal liver cell and tissues. Western blot analysis of MACC1 expression in THLE3 cells, PHH cells and two normal liver tissues. GAPDH was used as a loading control. (TIF) [file pone.0064235.s001.tif]
